# Functional Characterization of Date Palm Aquaporin Gene *PdPIP1;2* Confers Drought and Salinity Tolerance to Yeast and Arabidopsis

**DOI:** 10.3390/genes10050390

**Published:** 2019-05-22

**Authors:** Himanshu V. Patankar, Ibtisam Al-Harrasi, Rashid Al-Yahyai, Mahmoud W. Yaish

**Affiliations:** 1 Department of Biology, College of Sciences, Sultan Qaboos University, P.O. Box 36, Muscat 123, Oman; himanshu30@gmail.com (H.V.P.); i.alharrasi@gmail.com (I.A.-H.); 2 Department of Crop Sciences, College of Agricultural and Marine Sciences, Sultan Qaboos University, P.O. Box 34, Muscat 123, Oman; alyahyai@squ.edu.om

**Keywords:** aquaporins, abiotic stress, salinity, date palm, functional characterization

## Abstract

Recent studies on salinity tolerance in date palm revealed the discovery of salt-responsive genes including *PdPIP1;2*, a highly conserved aquaporin gene in plants, which was functionally characterized in this study to investigate its precise role in drought and salinity tolerance. Immunoblot assay showed a high level of PIP1 protein accumulation only in the leaves of date palm plants when grown under drought, an observation which may imply the involvement of PIP1;2 in CO_2_ uptake. Heterologous overexpression of *PdPIP1;2* in yeast (*Saccharomyces cerevisiae*) improved tolerance to salinity and oxidative stress. While, heterologous overexpression of *PdPIP1;2* in Arabidopsis had significantly (*p* < 0.05) increased biomass, chlorophyll content, and root length under drought and salinity. In addition, a significantly (*p* < 0.05) higher percentage of transgenic plants could be recovered by rewatering after drought stress, indicating the ability of the transgenic plants to maintain water and viability under drought. Transgenic plants under drought and salinity maintained significantly (*p* < 0.05) higher K^+^/Na^+^ ratios than wild type (WT) plants, an observation which may represent an efficient tolerance mechanism controlled by the transgene. Collectively, this study provided an insight on the mechanism by which *PdPIP1;2* conferred tolerance to salt and drought stresses in date palm.

## 1. Introduction

Date palm (*Phoenix dactylifera* L.) is a prime agriculture crop in the Arabian Peninsula and Northern Africa. Its nutritious fruits hold a socioeconomic status and the yield is the main source of income for some of the local populations [1]. Date palm is relatively tolerant to drought and salinity conditions, despite the fact that a large number of plants has been affected by a tremendous increase in soil salinity [2,3,4].

To understand different traits related to the ability of date palm to tolerate salinity, global differential transcriptome and methylome analysis of salt-stressed and non-stressed plants were previously conducted [5,6]. The transcriptomic study revealed a differential expression level of several gene homologues to those previously characterized in other plant species. To further understand the mechanism of salinity tolerance in date palm, the whole cDNA library was cloned and functionally characterized using yeast functional bioassay [7]. This study revealed the presence of 47 date palm genes which acquired salinity tolerance to yeast, including *PdPIP1;2*.

Plasma membrane intrinsic proteins (PIPs) are aquaporins belonging to a highly conserved large family of major intrinsic protein (MIP) in plants [8]. PIPs are divided into two phylogenetic groups: PIP1 and PIP2. The members of these two groups are known to interact with each other for the modulation of water balance [9], where they serve as water channels. Previous studies have also shown the involvement of these genes in ion exchange across the cellular membranes [10].

Several aquaporin PIP genes from various plants species are involved in abiotic stress tolerance mechanisms especially in the case of drought and salinity tolerance [11]. Abiotic stresses like drought and salinity causes water deficiency and alters the transmembrane water movement. Aquaporins are transmembrane water channels and their expression can regulate the transmembrane water movement. In addition, aquaporins function in transport of other ions and molecules, and hence, regulate a complex mechanism, which can be important especially in salinity stress [12,13]. A number of studies have shown that the overexpression of aquaporin in transgenic plants increases resistance to abiotic stresses. For example, the maize PIP2 aquaporin *MzPIP2;1* was involved in water uptake and transportation and improved drought and salinity tolerance of the transgenic Arabidopsis [14]. Overexpression of barley aquaporin gene *HvPIP2;5* in yeast and Arabidopsis confers salinity and osmotic stress tolerance [15]. In addition, transgenic Arabidopsis with the *Vicia faba VfPIP1* gene improved drought tolerance by inducing stomatal closure in order to prevent transpiration and therefore water loss [16]. In a study on the *VzPIP2;1* gene cloned from drought-tolerant vetiver grass (*Vetiveria zizanioides*), it was shown that the transgenic soybean exhibited a better performance than the wild type (WT) equivalent under drought stress when transformed using *Agrobacterium rhizogenes* [17].

Since PIP genes provided tolerance to abiotic stresses in other plant species, characterization of date palm *PdPIP1;2* may provide additional knowledge of mechanisms governing abiotic stress tolerance, as well as a basic tool toward producing salinity-tolerant date palms. Therefore, in this study, a PIP gene family from date palm was computationally analyzed and a member of this family (*PdPIP1;2*) was selected and functionally characterized in yeast and Arabidopsis.

Computational analysis revealed that the date palm genome encountered 40 aquaporin genes clustered into four phylogenetic groups including three copies of PIP1;2. In addition, the promotor of *PdPIP1;2* codes for a high number of transcription factors binding sites (TFBSs) concerning a potential function in abiotic stress tolerance. Overexpression of *PdPIP1;2* in yeast and Arabidopsis enhanced the growth of yeast in salinity and oxidative stresses, as well as enhanced drought and salinity tolerance in Arabidopsis. The study proposed that *PdPIP1;2* may facilitate water stress tolerance particularly in case of drought and salinity stresses in transgenic Arabidopsis, which will indeed provide valuable information relating to the abiotic stress tolerance in date palm. In addition, this study is the first of its kind which involves functionally characterizing a gene from the date palm genome.

## 2. Material and Methods

### 2.1. Computational Analysis of the Aquaporin Gene Family in Date Palm

To understand the relationship between the aquaporin gene family in date palm and other plant species, all the available aquaporin protein sequences of date palm and a small number of other selected *PdPIP1;2* sequence orthologs of different plants species were retrieved from the NCBI database (https://www.ncbi.nlm.nih.gov/) for computational analysis. The sequences were aligned using ClustalW algorithm [18], while the phylogenetic tree was constructed using the neighbor-joining method implemented within the Mega 7.0 software [19]. In addition, information regarding the putative regulatory sequences, located within the 2000 bp promotor region of the *PdPIP1;2* gene, was obtained using the PlantPAN 2 online software tool available from www.plantpan2.itps.ncku.edu.tw [20]. The results obtained using the Plant PAN 2 software were manually analyzed, and a figure representing abiotic stress-responsive transcription factors was generated accordingly.

### 2.2. Plant Growth Conditions, Protein Extraction, and Western Blot

Date palm (*P. dactylifera*) cultivar Khalas seeds were germinated and grown based on a previously described protocol [21]. Briefly, date palm seedlings were grown in 2-L pots containing potting mixture for two months in a greenhouse under controlled environmental conditions at 30 °C and natural sunlight. Plants were then divided into three groups for the control, salinity and drought treatments. Control and salinity group plants were watered with distilled water (electrical conductivity of 0.02 mS/m) and 300 mM NaCl solution, respectively. Drought stress was induced by withholding water for 14 days after two months of regular irrigation with distilled water.

Total protein was extracted from the date palm leaves and roots using previously described protocols [22,23], with minor modifications. Briefly, a 1:1 combination of Tris-buffer pH 8.0 and SDS buffer (2% SDS, 5% sucrose 5% β-mercaptoethanol, 0.1 M Tris-HCl, pH 8.0) was used for protein extraction. The total protein samples obtained from this extraction were quantified using the BCA Protein Assay Kit (Thermo Fisher Scientific, Rockford, Illinois, USA). The protein samples were loaded into the 12% TGX Stain-Free™ FastCast™ Acrylamide Kit (Bio-Rad Laboratories, Inc., Hercules, California, USA), then the fluorescent protein bands in the gel were quantified and normalized based on the total protein added in each lane using Image Lab 6.0.1 software (Bio-Rad, USA). The standard western blot protocol was performed for three biological replicates using 0.45 µm Immobilon-FL PVDF membrane (Merck Millipore, USA) and the Trans-Blot^®^ Turbo™ Transfer System (Bio-Rad, USA). Polyclonal anti-rabbit PIP1;2 primary antibody (catalogue number AS09 489, Agrisera, Vannas, Sweden) at 1:1000 dilution, and HRP-linked secondary anti-rabbit antibody (catalogue number AS09602, Agrisera) at a 1:1000 dilution was used in this experiment. The chemiluminescence signal on the immunoblot was detected using Clarity ECL (Bio-Rad, USA) and visualized using the ChemiDoc™ Touch Imaging System (Bio-Rad, USA). The immunoblot images were acquired and analyzed using Image Lab 6.0.1 software (Bio-Rad, USA). After developing the blots, the membranes were washed out from the antibodies, blocked using a milk solution, and incubated again with the polyclonal anti-rabbit actin (Agrisera, catalogue number AS13 2640) primary antibody which was used as the reference for the normalization of protein expression. The same type of secondary antibodies was used to detect the actin on the bolts.

### 2.3. Cloning and Functional Characterization of PdPIP1;2 in Yeast

The full cDNA sequence of *PdPIP1;2* gene was cloned into the yeast expression vector pYES-DEST52 (Thermo Fisher Scientific, USA), using site-specific recombination Gateway^®^ technology (Thermo Fisher Scientific, USA). The cDNA was cloned downstream of the *GAL1* promotor, which is induced by galactose. In a previous study, the WT yeast (*S. cerevisiae*) strain INVSc1 was used as functional bioassay [7]; therefore, in this study, the mutant salt-sensitive yeast (*S. cerevisiae*) strain BYT458 [24] (kindly provided by Prof. Hana Sychrova, Czech Republic) was selected for more accurate functional characterization experiments. Yeast genetic transformation was carried out using Yeastmaker™ Yeast Transformation System 2 (Clontech laboratories Inc., Mountain View, California, USA) following the manufacturer’s instructions. Transformant yeast (TY) colonies were selected based on the *URA1* auxotrophic selectable marker gene.

The ability of the transformed yeast cells with *PdPIP1;2* (TY) to tolerate different abiotic stresses, including salinity, drought, ionic stress and oxidative stresses, was tested against the transformed yeast cells with the pYES-DEST52 empty vector (EV) as the control, using yeast spot assay as previously described [7]. For the stress experiments, solid synthetic media (SSM) containing galactose were supplemented either with 300 mM NaCl (for salinity stress), 2% polyethylene glycol (PEG8000) (for drought stress), 10 mM LiCl (for lithium stress) or 3 mM H_2_O_2_ (for oxidative stress).

The growth rate of TY and EV cells was monitored by growing the cells in 20 mL liquid synthetic media (LSM) supplemented with 50 mM NaCl, incubated in a shaking incubator at 30 °C and agitated at 200 rpm. The optical density (OD) of the liquid culture was monitored every 12 h for three days.

### 2.4. Cloning of PdPIP1;2 in Binary Vector and Arabidopsis Genetic Transformation

*PdPIP1;2* cDNA was cloned into the binary plant expression vector pEarleyGate203 (TAIR stock ID: CD3-689), using site-specific recombination Gateway^®^ technology (Thermo Fisher Scientific, USA). The gene was cloned under the control of the *35S CaMV* constitutive in-plant promotor. First, the construct was genetically transformed into *Escherichia coli* cells for DNA amplification and then into the *Agrobacterium tumefaciens* LBA4404 strain by electroporation using the standard protocol.

To generate transgenic Arabidopsis lines, 45-day-old WT *Arabidopsis thaliana* Columbia (Col0) plants, which had reached the flowering stage, were transformed using the standard floral dip method [25]. To increase the transformation efficiency, the genetic transformation was repeated twice with a two-week interval. After the seeds had matured and dried, they were collected from the Arabidopsis plants, and positive transformants seeds (T0) were selected in soil by screening with the use of BASTA^®^ non-selective glyphosate herbicide solution at 0.01% (v/v) on the sixth and 10th days after seed germination. Selected positive transgenic plants were confirmed by PCR using *35S* promoter forward (5′-CAAGACCCTTCCTCTATATAAG-3′) and OSC terminator reverse (5′-CGCATATCTCATTAAAGCAG-3′) primers.

Self-fertilized seeds (T1) were collected from T0 plants, sown in the soil and screened for herbicide resistance using the same method. Transgenic T1 lines that showed mendelian pattern of segregation for the herbicide resistance were selected for further analysis. The second consecutive transgenic generation (T2) seeds were harvested from the positive T1 lines and transgenic homozygous plants were selected based on the results of PCR detection and herbicide resistance. The third consecutive transgenic homozygous lines (T3) were selected from the self-fertilized T2 plants. Three independent homozygous transgenic Arabidopsis lines from the third transgenic generation (T3) were selected for the following experiments.

### 2.5. Evaluation of the Stress Tolerance of Transgenic Arabidopsis Lines In Vitro

Arabidopsis seeds were planted on petri dishes containing half-strength Murashige and Skoog (MS) solid medium. The MS media plates were kept in a growth chamber under controlled environmental conditions of 22 °C, 70% relative humidity (RH), and a 16-h day/8-h night photoperiod. Three independent transgenic lines were tested for drought and salinity stress tolerance against WT Arabidopsis plants. Drought and salinity stress were implemented by supplying half-strength solid MS media with 150 mM mannitol and 100 mM NaCl, respectively. Initially, the seeds were germinated on half-strength MS media plates for four days. Further, the seedlings were transferred to the treatment and control plates and grown for an additional 14 days before the measurements were taken. The experiment was conducted using four technical and three biological replicates.

### 2.6. Evaluation of Stress Tolerance of the Transgenic Lines Grown in Soil

Three independent transgenic lines were tested for drought and salinity stress tolerance against WT Arabidopsis plants. Seeds were germinated on half-strength MS media for four days, then the seedlings were transferred to 0.5-L pots containing potting mixture soil, which has been previously watered to field capacity with distilled water. All the plants were grown in a growth chamber under controlled environmental conditions of 22 °C, 70% RH, and a 16-h day/8-h night photoperiod. The seedlings were given enough time to grow and acclimatize for 21 days before initiation of the stress treatments. Drought stress was applied by withholding water for 14 days, while salinity stress was applied by watering plants with 200 mM NaCl solution every three days for 14 consecutive days. Fourteen days after the initiation of drought treatment, plants were allowed to recover from drought by rewatering them with distilled water. The survival percentages of these plants were calculated after 48 h of the rewatering process. The soil moisture, temperature, and electrical conductivity were measured using EM50 ECH_2_O logger (Decagon Devices, Inc. Pullman, Washington, USA). Soil water potential was measured using a tensiometer (Irrometer, Riverside, California, USA).

### 2.7. Sodium (Na^+^) and Potassium (K^+^) Measurements in Yeast and Plants

TY and EV yeast strains were grown in 10 mL of LSM supplemented with galactose, or in the treatment media (LSM) supplemented with galactose and 25 mM NaCl for 48 h at 30 °C and shaken at 200 rpm in a shaking incubator. To extract Na^+^ and K^+^ from yeast cells, cells were collected in 2 -mL tubes by centrifugation at 5000 rpm for 5 min before being washed three times with sterile distilled water. Finally, the yeast cells were suspended in 1 mL sterile distilled water and boiled at 100 °C for 20 min in a water bath. After boiling the tubes, they were centrifuged at 12,000 rpm for 10 min, with the clear supernatant separated and diluted 10 times for Na^+^ and K^+^ measurements using Systronics Flame Photometer 128 (Systronics India Ltd. Ahmedabad, Gujarat, India) as previously described [26].

Arabidopsis plant samples were prepared by digesting the plant tissue using weak nitric acid as previously described by Munns et al. [27]. Briefly, plant samples were dried, weighted and digested in 10 mL of a 0.1 M nitric acid solution. Subsequently, the samples were kept at room temperature and shaken for two days at 100 rpm before being filtered using Whatman No. 1 filter paper and used for Na^+^ and K^+^ measurements. The Na^+^ and K^+^ concentrations were measured as previously mentioned in this study.

### 2.8. Measurement of Chlorophyll Content

Leaves were collected from the control and treated plants, while the total chlorophyll content was measured using the 80% acetone-based method as previously described [28]. The chlorophyll content was expressed as the µg/mg of fresh weight (FW) of the leaf tissue.

### 2.9. Statistical Analysis

One-way analysis of variance (ANOVA) and Tukey’s test was used to compare the statistically significant differences (*p* ≤ 0.05) between the mean of tested parameters. The statistical analysis was performed using IBM SPSS statistical package version 21 (IBM Corp. Armonk, NY, USA).

## 3. Results

### 3.1. Aquaporin is a Multigene Family in Date Palm

**Figure 1:**
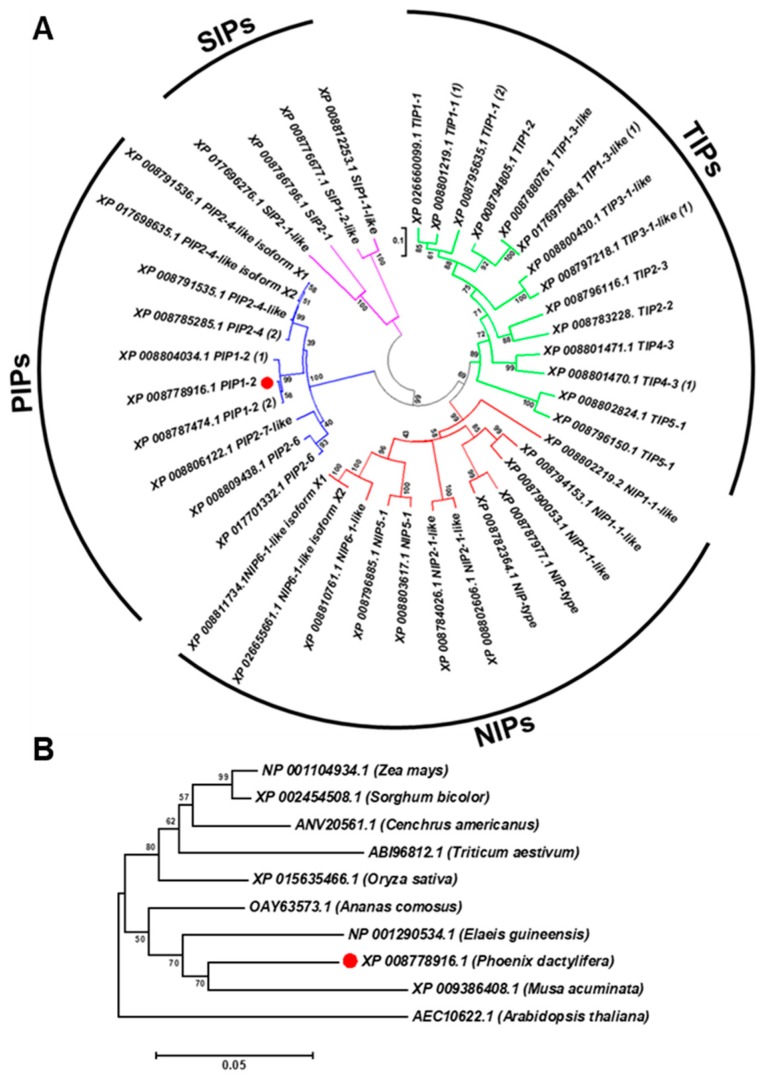
*PdPIP1;2* belongs to a large, conserved aquaporin family. The phylogenetic analysis of 40 date palm aquaporins (**A**) and the phylogenetic relationship between *PdPIP1;2* isoforms (marked by red circle) and aquaporins of other plant species (**B**). The phylogenetic trees were constructed using the amino acid sequences and the neighbor-joining method. Bootstrap values on the nodes represent percentages of 1000 repetitions.

Sequence analysis of the *PdPIP1;2* (GenBank accession number XM_008780694.2) revealed that the coding sequence of this gene is composed of 864 bp and codes for 287 amino acid protein sequences with a predicted molecular weight of 30.84 kDa and a theoretical isoelectric point of 8.60. The aquaporin gene family was analyzed in order to ascertain the structure of this gene family in date palm and its relationship with aquaporins of different plant species. The results showed that the aquaporin family in the date palm genome was composed of 40 members, divided into four main clusters of subfamilies: PIPs, SIPs, NIPs and TIPs. The family included three copies of *PdPIP1;2*, which were clustered together within the same group. The analysis also showed that *PdPIP1;2* is similar to *PdPIP2;4* as the two groups cluster together in the same branch (Figure 1A).

**Figure 2:**
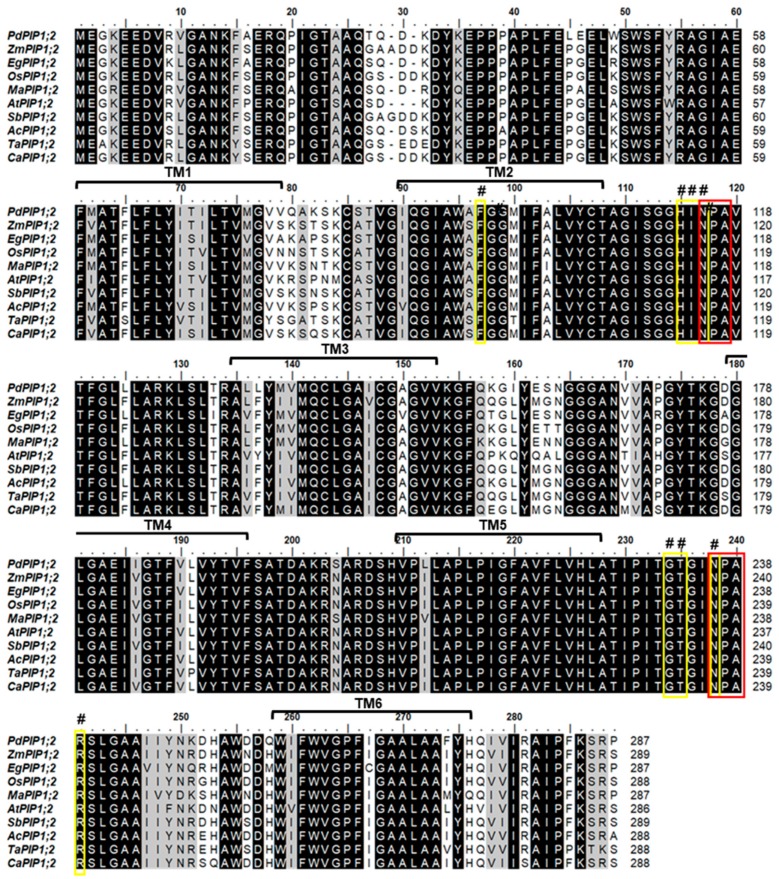
Mapping of conserved domains on *PdPIP1;2*. The multiple sequence alignment of the deduced amino acid sequence of *PdPIP1;2* and other PIP1;2 isoforms from other plant species. The dark-colored highlight shows the identical/conserved regions and the gray highlights show similar regions with a one amino acid difference. The yellow boxes with the (#) sign refer to the eight amphipathic channel residues while the red boxes show the conserved Asn-Pro-Aln motif signature of major intrinsic protein (MIP) family. Six transmembrane ™ domains are represented on the sequence alignment.

Phylogenetic analysis of the protein sequence revealed that the date palm PIP1;2 gene is closely related to banana (*Musa acuminate*) and African oil palm (*Elaeis guineensis*) along with pineapple (*Ananus comosus*). Further, the PIP1;2 orthologous genes identified from monocots species were clustered in one group whereas the only dicot PIP1;2 of Arabidopsis used in this analysis was branched out from the group, indicating a lower degree of similarity with *PdPIP1;2* and those PIP1;2 of other monocots (Figure 1B). Multiple sequence alignment of the deduced amino acid sequence of PIP1;2, identified from different plant species, showed that more than 80% of conserved regions amongst the sequences included two signatures of the asparagine-proline-alanine (NPA) motifs and eight individual amino acid residues, which may have formed the amphipathic channel in these proteins. In addition, the protein sequences included six putative transmembrane (TM) domains, which usually code for a bundle of six transmembrane α-helices [29] (Figure 2).

### 3.2. Promoter Sequence Analysis Revealed Abiotic Transcription Binding Sites in PdPIP1;2

**Figure 3:**
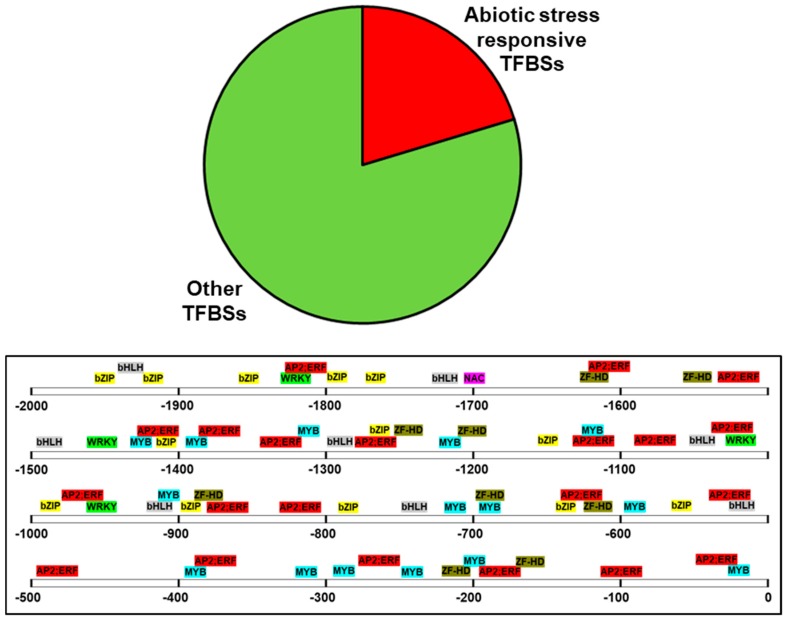
Sequence analysis of the putative promoter region of *PdPIP1;2*. The figure shows the distribution of abiotic stress-responsive transcription factors binding sites (TFBSs), which represent 20% of the total TFBSs predicted within the 2000 bp of the putative promotor.

In order to obtain an idea about the environmental factors that may induce the expression of *PdPIP1;2*, the 2000 bp sequence upstream of the coding region, which represents a putative promotor region, was analyzed using bioinformatics tools. The analysis revealed the presence of 954 putative TFBSs on the (+) DNA strand, 20% of which are known to be involved in abiotic stresses, such as bZIP, ZF-HD, NAC, bHLH MYB, WRKY, AP2; ERF was previously found in drought- and salinity-inducible genes [30,31,32,33]. These types of TFBS were found in abundance close to the ATG start codon of the *PdPIP1;2* (Figure 3).

### 3.3. Drought Stress Increased PdPIP1;2 Protein Accumulation in Date Palm Leaves

**Figure 4:**
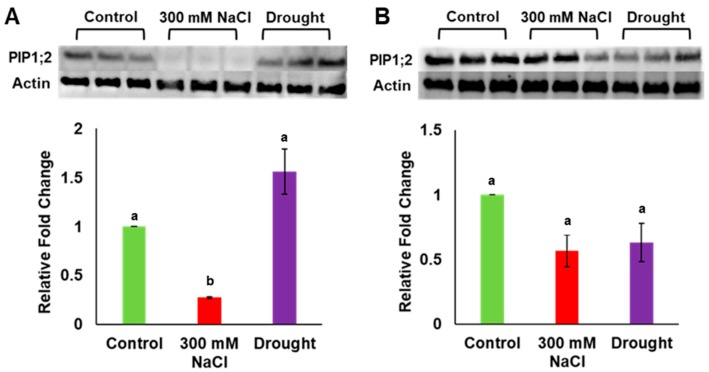
Analysis of *PdPIP1;2* protein expression in leaf and root tissues under drought and salinity conditions. Protein immunoblot image showing the levels of *PdPIP1;2* protein accumulation in leaves (**A**) and roots (**B**) of date palm seedlings, grown under control, salinity and drought conditions of three biological replicates. The bar graph shows the mean of the relative fold change of accumulation (± SE, *n* = 3) in salinity and drought stress, compared to control conditions. Asterisks indicate a significant difference at *p* < 0.05.

Gene expression analysis previously showed that *PdPIP1;2* was highly expressed in leaf and root tissues of date palm under salinity [7]. In order to verify the amount of expression at the protein level, total protein was extracted from the leaf and the root tissues of date palm seedlings grown under drought, salinity and control conditions, and the level of protein accumulation was determined in both tissue types using the western blot technique. It was observed that the level of PIP1;2 accumulation was significantly (*p* ≤ 0.05) lower than for the control in the date palm leaf samples when grown under salinity; however, this level was slightly higher than for the control in the same tissue under drought stress (Figure 4A, Supplementary Figure S1a). Surprisingly, the level of PIP1;2 protein was significantly (*p* ≤ 0.05) lower than for the control in the root tissues when plants were grown under both salinity and drought conditions (Figure 4B, Supplementary Figure S1a). In addition, the antibodies used in this analysis could detect other isoforms of PdPIP1 proteins, which showed a similar trend of accumulation in the plant tissues under the different environmental conditions (Supplementary Figure S1b).

### 3.4. Overexpression of PdPIP1;2 Enhanced Salinity Tolerance in Yeast

**Figure 5:**
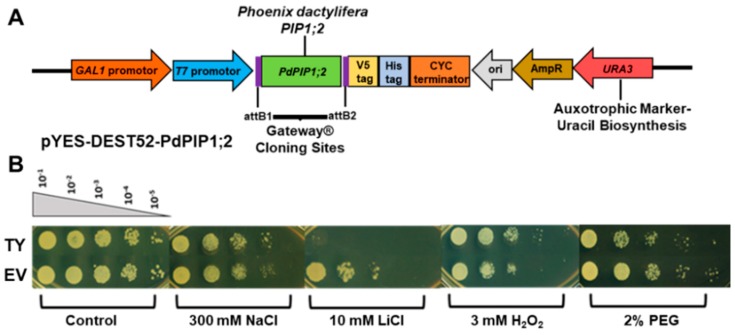
Schematic representation of cloned *PdPIP1;2* gene in pYES-DEST52 plasmid for overexpression in yeast (**A**). The effect of *PdPIP1;2* transgene on the growth of yeast cells on solid culture. Yeast spot assay used to test the relative tolerance of transformant yeast (TY) and empty vector (EV) cells when grown under control and other different abiotic stress conditions (**B**).

**Figure 6:**
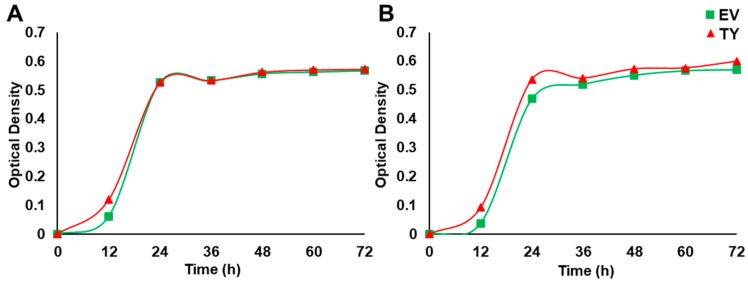
The effect of *PdPIP1;2* transgene on the growth of yeast cells in liquid culture. Yeast liquid culture assay used to test the relative tolerance of TY and EV cells grown under control (**A**) and 50 mM NaCl stress (**B**) conditions. The line represents the mean of the growth curves of yeast (± SE, *n* = 3).

Yeast cells were used in order to obtain a basic and relatively quick impression about the effect of *PdPIP1;2* on salt tolerance in eukaryotes. Spot assay was employed using a mutant salt-sensitive yeast strain transformed with pYES-DEST52-PIP1;2 (Figure 5A) in order to clearly verify the effect of *PdPIP1;2* on the TY cells when grown under salinity. Different concentrations of TY cells, along with cells transformed with an EV, were used in spot assay on SSM, where they showed different responses based on the abiotic stress used in the experiment. The TY and EV cells showed uniform growth on control SMM plates, whereas, when these plates were supplemented with 300 mM NaCl, the TY cells showed a better growth pattern than EV cells. TY cells failed to grow on SMM plates supplemented with LiCl; however, EV cells were able to survive. On the other hand, under oxidative stress, TY cells showed a better growth rate than EV cells. Drought tolerance, using yeast spot assay and PEG, revealed that TY cells were able to grow slower than in the case of EV cells (Figure 5B). In order to obtain numerical values for the growth rates during the different growth stages of yeast cells under salinity, the yeast strains were tested in LSM liquid culture supplemented with galactose and 50 mM NaCl. The results showed that the TY cells had a slightly higher growth rate than the EV cells, albeit only during the exponential growth phase (Figure 6).

**Figure 7:**
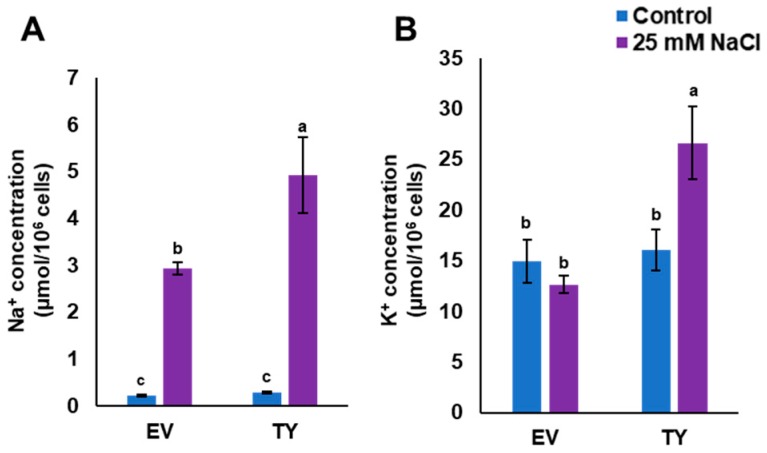
Accumulation of Na^+^ and K^+^ in transgenic yeast cells. Na^+^ (**A**) and K^+^ (**B**) concentrations in TY or EV when grown in liquid synthetic media (LSM) (control) and LSM supplemented with 25 mM NaCl as salinity stress conditions. The bars represent the mean concentration of Na^+^ and K^+^ (± SE, *n* = 3), while the asterisks indicate a significant difference to wild type (WT) at *p* < 0.05.

In order to monitor Na^+^ and K^+^ uptake, TY and EV cells were grown in LSM or in LSM supplemented with 25 mM NaCl. This concentration of NaCl was used because it is not a lethal concentration for yeast cells. The results showed that Na^+^ concentration in both yeast strains was low when the cells were grown in the control medium, whereas the Na^+^ concentration in TY cells was significantly (*p* < 0.05) higher than the Na^+^ concentration in EV cells when grown in 25 mM NaCl medium. Interestingly, the concentration of K^+^ in TY cells was increased by approximately two times when cells were grown in LSM supplemented with 25 mM NaCl; however, the K^+^ concentration in EV cells was similar when grown in both control and salt stress media (Figure 7).

### 3.5. PdPIP1;2 Confers Stress Tolerance to Arabidopsis In Vitro

**Figure 8:**
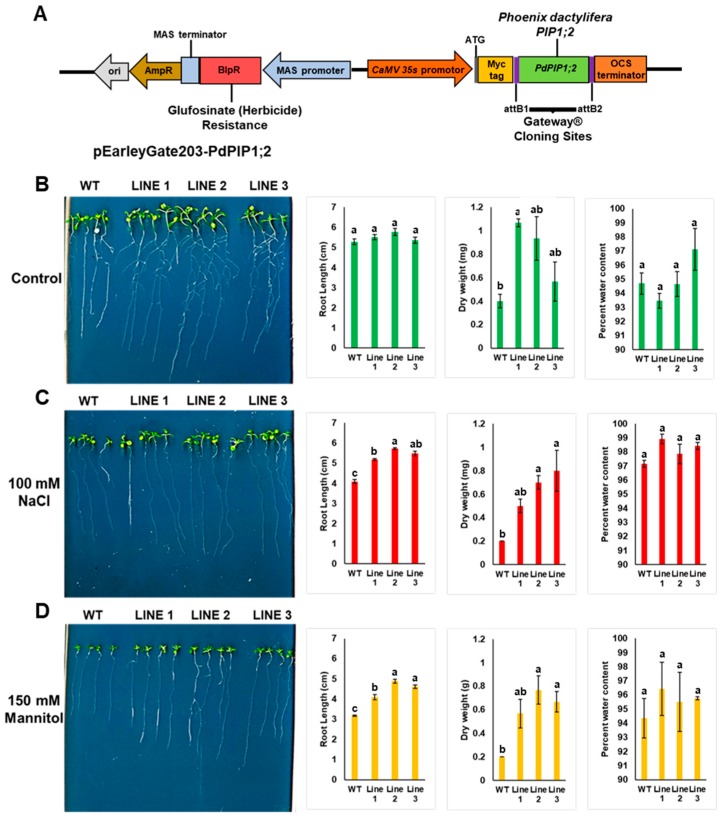
Heterologous overexpression of *PdPIP1;2* enhanced drought and salt tolerance in Arabidopsis. Schematic representation of cloned *PdPIP1;2* gene in pEarleyGate203 plasmid for overexpression in Arabidopsis (**A**) The growth pattern, root length, dry weight and water content percentage measurements in WT and transgenic Arabidopsis lines with *PdPIP1;2* grown in plane control (**B**), 100 mM NaCl (**C**), or 150 mM mannitol media (**D**) for 14 days. The length of the main root was measured manually using a standard centimeter scale. The bars represent either the mean root length in cm or dry weight in mg (± SE, *n* = 3), while the asterisks indicate a significant difference to WT at *p* < 0.05.

Heterologous overexpression of a *PdPIP1;2* gene in Arabidopsis is an important step towards determining the role of this gene in drought and salt tolerance in plants; therefore, *PdPIP1;2* was cloned into a binary vector under the control of the constitutive *35S CaMV* promoter (Figure 8A) and genetically transformed to Arabidopsis, while the transgenic lines were tested for their performance under control, drought and salinity conditions. The results showed the presence of a relatively high number of transgenic lines (T0), as indicated by herbicide screening and subsequently genotyping by PCR, revealing a clear product in agarose gel electrophoresis (Supplementary Figure S2).

Three independent homozygous lines from (T3) were selected for further analysis. WT and transgenic Arabidopsis seedlings were grown either on plain MS agar media plates as a control treatment, or on the same medium supplemented with NaCl or mannitol for salt or drought treatments, respectively. It was observed that WT and transgenic lines showed similar germination times and rates when grown under the same environmental conditions. The results also showed that the transgenic lines grown on control medium plates had a greater pattern of root branching than in the case of WT plants, yet all the plants were healthy. The roots of the transgenic plants were significantly (*p* < 0.05) longer than those of WT plants when grown under salinity; however, a unique pattern of root branching was not observed under salt stress (Figure 8 and Supplementary Figure S3). In addition, it was noted that transgenic lines grown under salinity had larger leaves. While fresh weight was slightly higher in the transgenic plants, the dry weights of the plantlets were significantly (*p* < 0.05) higher than those of WT plants when grown under drought or salinity (Figure 8). Transgenic plants grown under mannitol drought stress showed a stronger growth vigor in terms of root length and foliar growth in comparison to WT plants. The root system of the transgenic lines also showed more branches than WT lines under drought stress (Figure 8).

**Figure 9:**
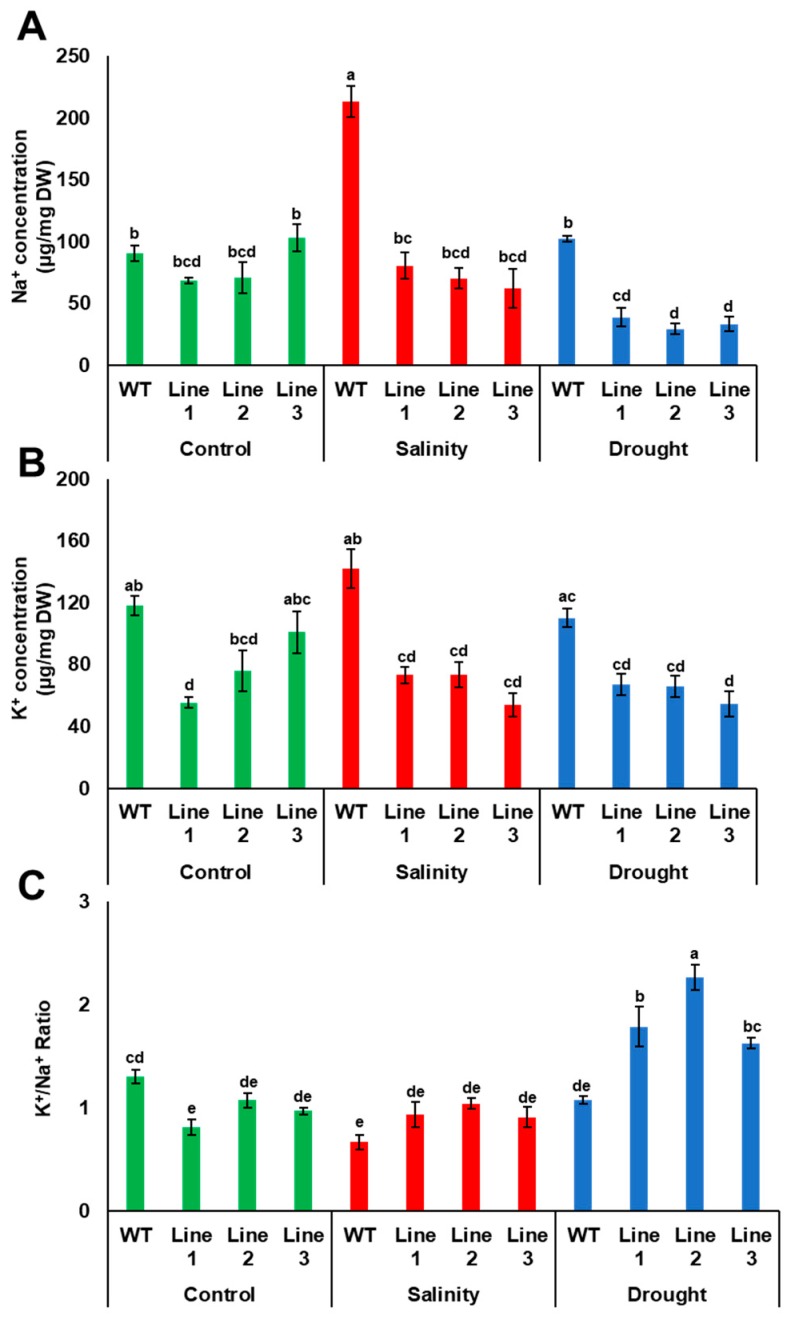
Accumulation of Na^+^ and K^+^ in transgenic Arabidopsis plants. Na^+^ (**A**) and K^+^ (**B**) concentrations and the K^+^/Na^+^ ratio (**C**) in WT and transgenic Arabidopsis lines with *PdPIP1;2* subjected to drought (150 mM mannitol) and salinity (100 mM NaCl) on half-strength MS medium plates. The bars represent the mean concentrations of Na^+^ and K^+^ (± SE, *n* = 3), while the asterisks indicate a significant difference to WT at *p* < 0.05.

As Na^+^ and K^+^ concentrations are important salinity tolerance factors in plants, the concentration of these ions was measured in transgenic Arabidopsis plants grown under both drought and salinity conditions. The results showed that Na^+^ concentration was similar in both transgenic and WT plants; however, K^+^ concentration was significantly (*p* < 0.05) lower in two transgenic lines, compared to WT plants when grown under control medium conditions. Under drought, Na^+^ and K^+^ uptake by transgenic plants were reduced compared to WT plants. The reduction in Na^+^ uptake by transgenic plants during drought was reflected in the K^+^/Na^+^ ratio, in which the transgenic plants had a significantly (*p* < 0.05) higher K^+^/Na^+^ ratio compared to WT plants. Under salinity, the concentration of Na^+^ in WT plants was significantly (*p* < 0.05) higher than in transgenic plants; however, the concentration of K^+^ was lower in transgenic than in WT plants, thus maintaining a relatively high K^+^/Na^+^ in the transgenic plants (Figure 9).

### 3.6. PdPIP1;2 Confers Stress Tolerance on Arabidopsis Grown in Soil

**Figure 10:**
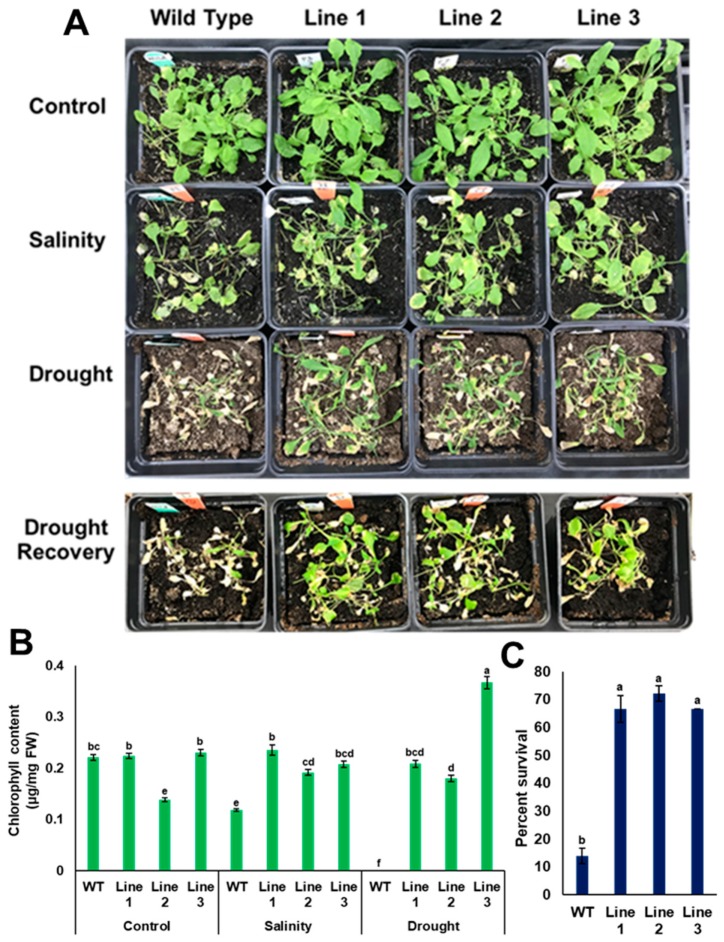
Heterologous overexpression of *PdPIP1;2* enhanced tolerance to salinity and drought recovery in Arabidopsis. Tolerance (**A**) and total chlorophyll content (**B**) of WT and transgenic Arabidopsis lines in the case of salinity (200 mM NaCl), drought and drought recovery. Survival percentages of the plants rewatered and recovered from drought treatment (**C**). The bars represent the mean (± SE, *n* = 3), while the asterisks indicate a significant difference to WT at *p* < 0.05.

Since the results obtained from transgenic plants grown on MS media in vitro may differ from transgenic plants grown naturally in soil, such plants were grown in potting mixture soil, then tested for their ability to sustain drought and salinity. For the drought experiment, water was withheld for two successive weeks before taking the measurements. The results showed that the soil moisture content at the end of the experiment was 0.002 m^3^/m^3^ and the electrical conductivity was 0.5 mS/m (Supplementary Figure S4). Additionally, soil water potential of the dry soil was about 60 kPa as measured by the tensiometer. It was observed that transgenic lines with *PdPIP1;2* showed a better performance than the control plants as indicated by the viability of the plants, while the chlorophyll content was significantly (*p* < 0.05) higher compared to WT plants after drought stress for the same period of time (Figure 10B). In addition, transgenic lines exhibited significantly (*p* < 0.05) higher recovery and survival percentages due to drought than WT lines after rewatering with distilled water (Figure 10A,C). For example, about 65% of transgenic plants were recoverable, whereas only about 10% of the WT plants were recoverable in 48 h (Figure 10C).

Transgenic plants with *PdPIP1;2* were stressed with salinity, together with WT Arabidopsis plants. At the end of the treatment, the EC of salt-treated and control soils was 5.5 and 0.47 mS/m, respectively (Supplementary Figure S4). The transgenic lines treated with salinity showed a better viability in comparison to WT lines (Figure 10A). In addition, transgenic lines exhibited a significantly (*p* < 0.05) higher level of chlorophyll than WT lines (Figure 10B).

## 4. Discussion

Date palm is a relatively drought and salinity tolerant plant; however, the key genes involved in the tolerance mechanism remain unknown. Understanding the mechanism behind tolerance is a critical factor, knowledge of which is required for the further enhancement of tolerance in plants [34]. In our efforts to understand the salinity tolerance mechanism, we explored the methylome [6], transcriptome [5], microbiome [35,36,37] and activity of some enzymatic and non-enzymatic antioxidants in date palm [21,38]. In addition, a date palm cDNA library was previously constructed and screened in yeast to identify genes involved in salinity tolerance using the functional bioassay approach [7].

*PdPIP1;2* gene, which is coding for aquaporin protein, was among those genes with enhanced salinity tolerance in WT yeast. The aquaporin of date palm, as characterized in this study, has a conservative sequence at the protein level and encodes potential abiotic stress-responsive TFBSs in the putative regulatory sequence. The phylogeny analysis and the multiple sequence alignment carried out in this study showed high sequence similarity between *PdPIP1;2* and other PIP1;2 isoforms from different plant species. This is consistent with the notion that PIP1 proteins are highly conserved amongst different plant species [39]. The promoter analysis of *PdPIP1;2* revealed the presence of a large number of abiotic stress-related TFBSs. Some of the predominant stress-related transcriptions factors were MYB, WRKY, AP2. Meanwhile, some previous studies have reported on the involvement of ERF and bZIP in plant abiotic stress responses [40,41,42,43]; therefore, the presence of a large number of abiotic stress-related TFBSs in *PdPIP1;2* promotor may indicate the involvement of this gene in abiotic stress tolerance mechanisms [44].

*PIP1;2* is highly expressed in the leaf and root tissues of date palm seedings when grown under salinity [7]; however, the immunoblot results of this study showed a decrease in the accumulation of *PdPIP1;2* protein in leaves and root tissue due to salinity stress (Figure 4). This discrepancy in mRNA and protein expression reveals that various other factors may be involved in the translation and post-translational modification processes, as well as the stability of proteins under salinity [45]. In addition, *PdPIP1;2* protein was accumulated in a lower amount in the root tissues in response to drought. Consistent with this observation, a previous study exhibited that PIP1 of Arabidopsis was downregulated in response to drought [46]. The downregulation of *PdPIP1;2* in roots of date palm may suggest a strategy used by the plants in which the cell is saving energy by reducing the amount of proteins involved in water transport, as the availability of water under drought stress is highly limited. On the other hand, *PdPIP1;2* protein was accumulated in a higher amount, compared to the control, in leaf tissues when date palm seedlings were exposed to drought conditions (Figure 4). This observation may support the notion that PIPs are also involved in CO_2_ conductance and assimilation. In fact, plants under drought stress try to reduce water loss through transpiration and thus keep stomata closed; therefore, overexpression of PIP1;2 in leaves could be a good strategy to balance CO_2_ uptake in this situation and in turn maintain the activity of the photosynthesis machinery [47]. A similar observation regarding the reduction in stomatal conductance and the increase in the internal CO_2_ was previously found in date palm due to reduction in water availability [48]. It was previously shown that Arabidopsis aquaporin *AtPIP1;2* is involved in cellular CO_2_ uptake and therefore enhances photosynthesis [47]. Similarly, overexpression of *HvPIP1;2* in rice enhances CO_2_ conductance and assimilation but transgenic plants are sensitive to salinity stress [49].

As a first step towards the functional characterization of *PdPIP1;2*, the gene was expressed in a salt-sensitive yeast strain, then tested for various types of abiotic stress. When exposed to salinity stress, these TY cells behaved slightly better than EV cells (Figure 5B). This observation could suggest the involvement of the *PdPIP1;2* gene in enhancing NaCl stress tolerance in this system; however, when exposed to LiCl stress, they exhibited extremely weak growth, whereas the EV control cells managed to grow under this type of ionic stress (Figure 5B). This finding could be due to the ability of *PdPIP1;2* to facilitate lithium ion uptake, which could cause lithium toxicity for the yeast cells as aquaporins are generally known to be involved in unspecific ion transportation across the membrane [50].

The quantification of Na^+^ and K^+^ in stressed yeast cells showed that TY cells had a better Na^+^ and K^+^ management system than in the case of the EV cells, meaning that the system can maintain a high level of K^+^ when grown under NaCl stress (Figure 7). This may imply that *PdPIP1;2* may directly or indirectly counterbalance the high Na^+^ by increasing K^+^ uptake under salt stress in yeast, which can be a feature mechanism for salinity tolerance in date palm. Nevertheless, *PIP1;2* is not known to be coding for an ionic channel.

Three independent transgenic Arabidopsis lines with *PdPIP1;2* were tested for their ability to tolerate drought and salinity stresses in MS media, where they revealed an enhanced pattern of root branching under control conditions; however, under drought and salinity, the performance of transgenic seedlings was improved only in terms of the main root elongation and the biomass. This observation may suggest the presence of an improved water management system through the roots as a result of the transgene. A previous study on date palm aquaporins showed that *PdPIP1;1* and *PdPIP1;3* are involved in the early stages of drought response in the roots of date palm [51].

Transgenic plants grown in soil were able to maintain growth vigor and tolerate salt and drought stresses more than WT plants (Figure 10). In addition, drought-stressed transgenic plants were able to recover after rewatering. Drought recovery may indicate that *PdPIP1;2* is involved in maintaining water usage under drought; therefore, this protein helps plants to tolerate stress for a longer time. Similarly, it was previously shown that the aquaporin genes *OsPIP1;2* and *OsPIP2;1* are involved in drought tolerance in rice as they are induced under water-deficient stress conditions [52]. Meanwhile, the overexpression of *Simmondsia chinensis* PIP1 enhances drought and salinity tolerance in Arabidopsis by reducing membrane damage and improving osmotic adjustments [53], and the overexpression of the PIP2;1 gene from drought-tolerant *Festuca arundinacea* perennial grass enhanced growth and development in Arabidopsis [54].

The K^+^/Na^+^ ratios were relatively higher in the transgenic lines than in the WT lines when the plants were grown under drought and salinity (Figure 9C). This observation could describe a strategy used by plants to overcome drought and salinity. Similarly, a previous functional study on the banana aquaporin *MaPIP1;1* demonstrated that transgenic Arabidopsis plants with *MaPIP1;1* were more tolerant to drought and salinity than WT plants due to a reduction in the membrane injury and the maintenance of a high K^+^/Na^+^ ratio [55].

Aquaporins play important roles in the regulation of plant water relations and abiotic stress responses, as it was previously shown that 35 aquaporin homologs from Arabidopsis were upregulated in response to drought stress [46]. In addition, there was a significant reduction in protoplast water permeability of Arabidopsis knockout mutants of *AtPIP1;2* and *AtPIP2;2* genes [56].

## 5. Conclusions

We have proven in the present study that the overexpression of one of the date palm aquaporins (*PdPIP1;2*) confers tolerance to salinity and oxidative stress in yeast, whereas it renders yeast sensitive to the LiCl. This observation suggests an increase in the membrane permeability to Na^+^, K^+^, and possibly other ions in yeast. Similarly, heterologous overexpression of *PdPIP1;2* improves the tolerance in Arabidopsis by maintaining a balanced K^+^/Na^+^ ratio and water content under drought and salinity. However, deciphering the specific role of PdPIP1 in these processes requires further investigation. The increase in the accumulation of PdPIP1 proteins under drought in date palm leaves could have a role in CO_2_ uptake, which is required for the maintenance of the photosynthesis system. This is the first study aimed at functionally characterizing a salt-responsive gene from date palm.

## Supplementary Materials

The following are available online at https://www.mdpi.com/2073-4425/10/5/390/s1, Figure S1a: The immunoblot analysis of PdPIP1;2 protein expression in leaf and root tissues under drought and salinity conditions, Figure S1b: The figure shows the normalized protein accumulation of the probable multimers of the PIP1 in leaf (A) and root (B) tissue detected on the immunoblot as seen in supplementary Figure S1A, Figure S2: Genotyping of transgenic Arabidopsis plants used to confirm the presence of the *PdPIP1;2* gene, using 35S forward and OSC terminator reverse primers in the PCR, Figure S3: The total root length (A) and the total root surface area (B) measured using WinRhizo root scanning system, Figure S4: The mean moisture percentage, electrical conductivity (EC) and temperature of the soil measured on the 14th day of the stress treatment represented as a bar graph.
