# Supplementary material for: Functional Characterization of Date Palm Aquaporin Gene PdPIP1;2 Confers Drought and Salinity Tolerance to Yeast and Arabidopsis
Source: Genes (Basel). 2019 May 22;10(5):390. doi: 10.3390/genes10050390 (PMC6562508; doi:10.3390/genes10050390)
Supplement: Supplementary file 1 [file genes-10-00390-s001.pdf]

The original images of the western bolts of Fig. 4a. The target PIPs1-2 protein has a molecular weight of 30.2 kDa, whereas the molecular weight of actin is about 45 kDa.

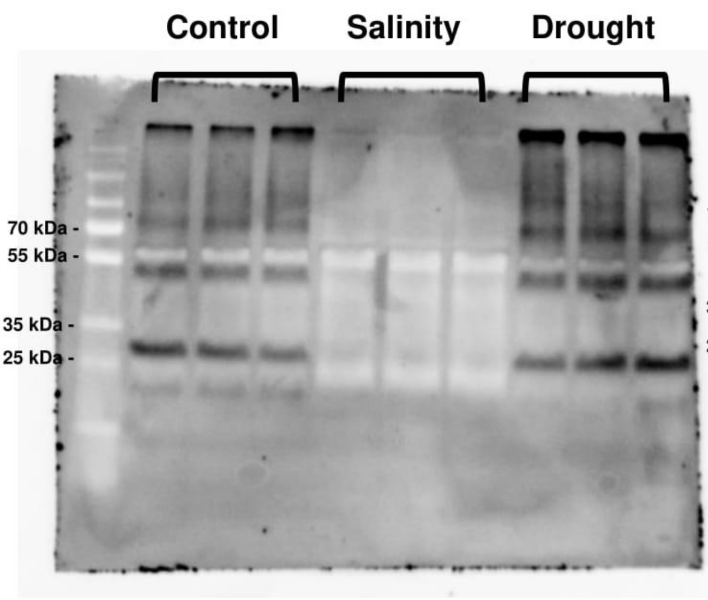

Leaf Protein – PIP1-2 Antibody

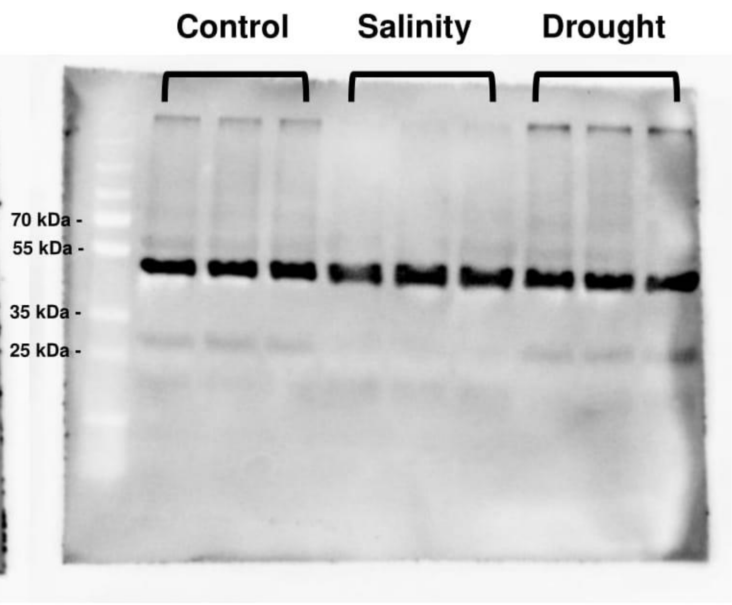

Leaf Protein – Actin Antibody

The original images of the western bolts of Fig. 4b. The target PIPs1-2 protein has a molecular weight of 30.2 kDa, whereas the molecular weight of actin is about 45 kDa.

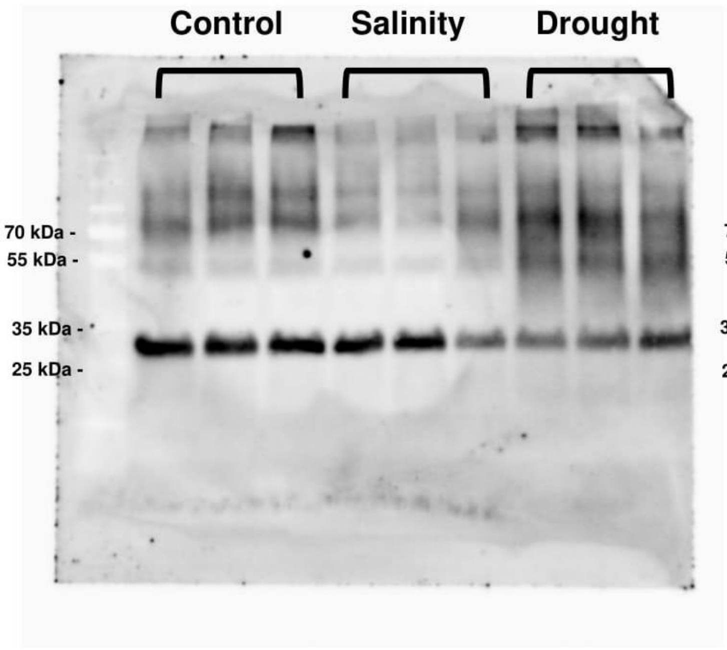

Root Protein – PIP1-2 Antibody

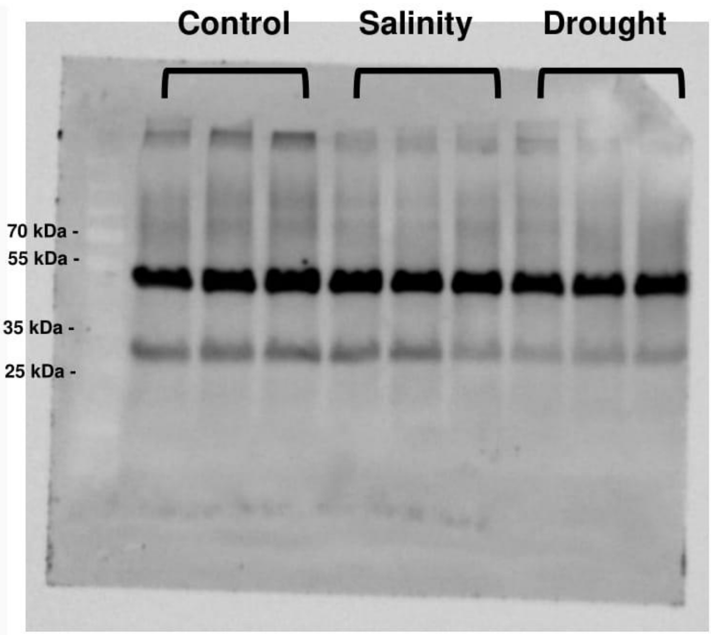

Root Protein – Actin Antibody

Supplementary Figure S1a: The immunoblot analysis of PdPIP1;2 protein expression in leaf and root tissues under drought and salinity conditions. The figure shows the original images of the western bolts presented in Figure 4.

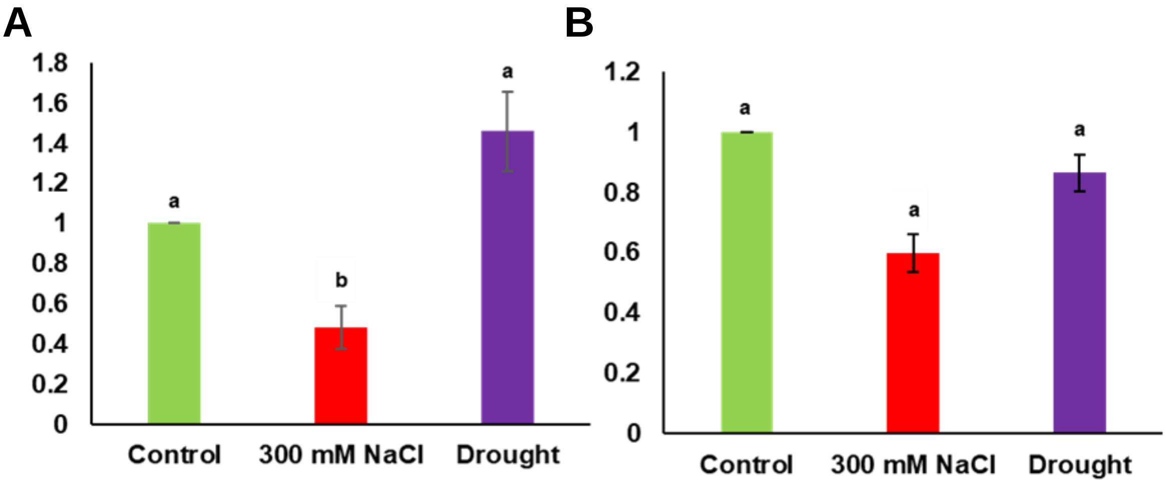

Supplementary Figure S1b. The figure shows the normalized protein accumulation of the probable multimers of the PIP1 in leaf (A) and root (B) tissue detected on the immunoblot as seen in supplementary Figure S1A.

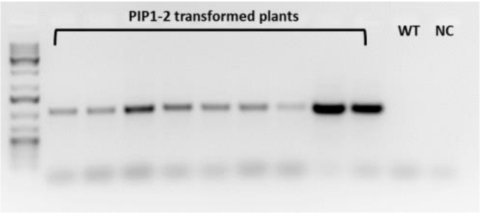

Supplementary Figure S2: Genotyping of transgenic Arabidopsis plants used to confirm the presence of the PdPIP1;2 gene, using 35S forward and OSC terminator reverse primers in the PCR. Genomic DNA extracted from WT plants or no DNA (NC) was used in the PCR as a negative control.

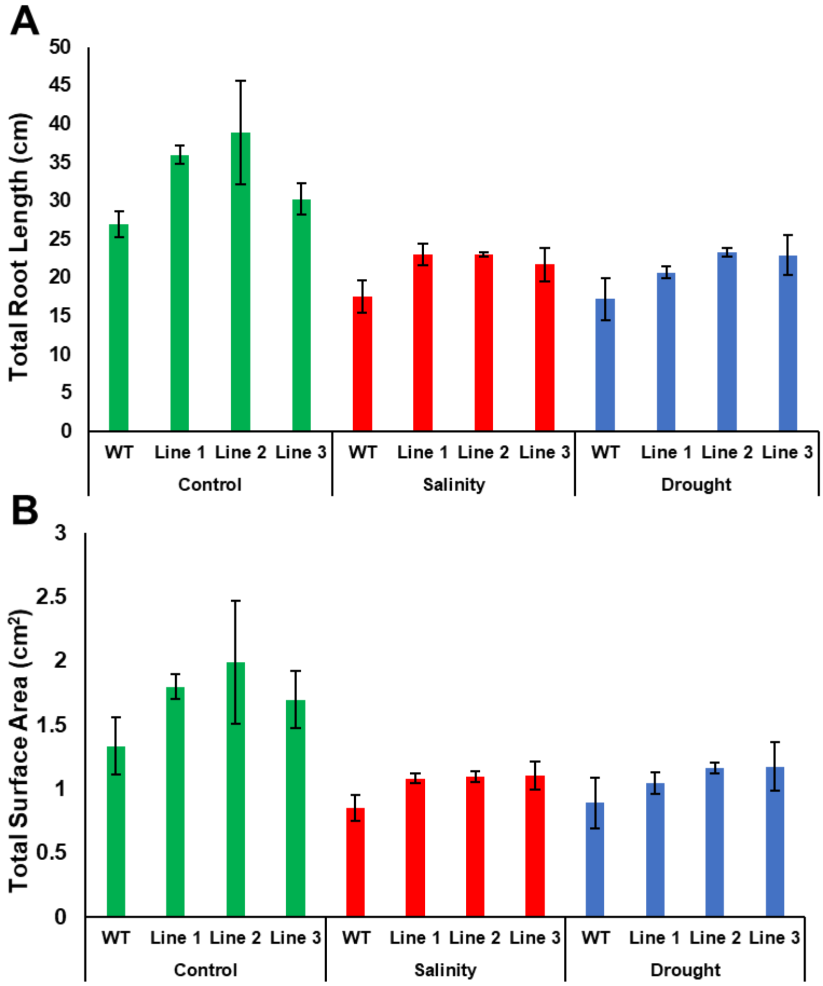

Supplementary Figure S3: The total root length (A) and the total root surface area (B) measured using WinRhizo root scanning system (Reagent Instruments, Canada) (± SE, n = 3).

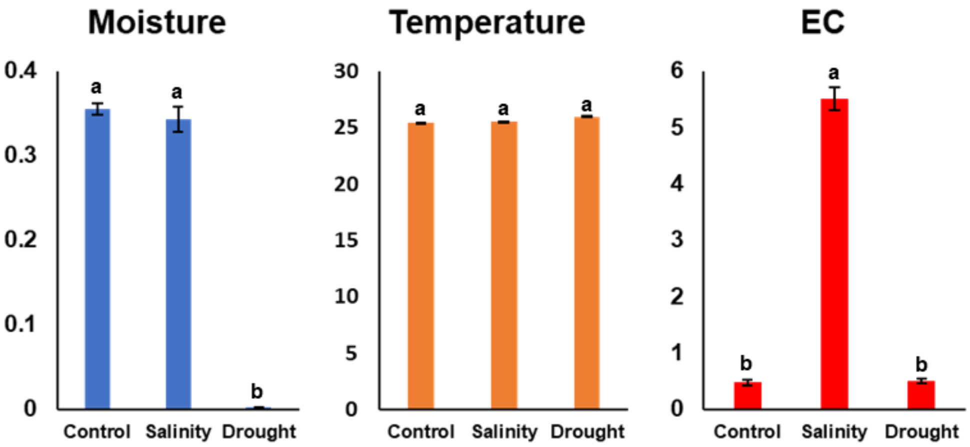

Supplementary Figure S4: The mean moisture percentage, electrical conductivity (EC) and temperature of the soil measured on the 14th day of the stress treatment represented as a bar graph (± SE, n = 12).
